# Medical Items and News of Interest to the Physician

**Published:** 1872-07

**Authors:** 


### For the Use of Physicians.

CULLED FROM THE STANDARD MEDICAL JOURNALS OF THE WORLD.

NOTE.—These formulæ are intended only for the regular practitioner of medicine, and should be employed only by him, or under his immediate supervision.

### Antidote for Carbolic Acid.

A strong solution of saccharate of lime, is a reliable antidote against the poison of carbolic acid, when by accident taken internally.—*Druggists Circular.*

### Incontinence of Urine in Children.

Mr. Holmes Coote recommends for this intractable affection the administration of creasote in one minim doses, three times daily, combined with assafœtida and rhubarb pill, of each two grains.

### Sloughing Ulcers.

Take two ounces of starch and mix with six ounces of boiling water, to form a jelly, then add, before cold, half an ounce of tincture of iodine. Apply the poultice to the sloughing surface.—*Medical Record.*

### Peritonitis.

The oxalate of potash, in the treatment of peritonitis, is recommended in the *Jour. de Med. et de Chir.* Small doses in mucilage, repeated every hour, having produced good results in three cases of puerperal origin.

### Traumatic Tetanus.

Clinton Cushing, M. D., Oakland, Cal., (*Pacific Med. & Surg. Jour.*), has successfully treated a case of this malady, under repeated doses of 1-12 grain of the English extract of calibar bean, and 15 grains of the hydrate of chloral.

### Sulphate of Iron in Suppuration.

A child, burned all over, was lately brought into the hospital. The suppuration from the wound was so profuse and offensive, that the ward in which he lay was almost uninhabitable.

He was placed in a bath containing two handsful of sulphate of iron. The cessation of pain was almost immediate ; after repeating the bath twice a day for fifteen or twenty minutes at a time, the suppuration moderated, the fetid odor disappeared, and the patient rapidly recovered. —*Boston Jour. Chemistry.*

**Vomiting of Pregnancy.**

Dr. A. F. Pattee, Boston, speaks very highly of the use of syrup of the hypophosphite of lime, in obstinate cases of vomiting in pregnancy. The dose ranges from ʒ j to ℥ ss, given with the food, three or four times a day.—*Medical Record.*

---

**Tincture of Iodine in Vomiting.**

Schneider of Offenburg, (*The Doctor*, April, 1872,) administered tincture of iodine in doses of ten drops on sugar, thrice daily to a patient who was troubled with salivation and vomiting after intermittent fever. Although the vomiting had proved rebellious to all other treatment, it yielded to tincture of iodine.

---

**Tenia.**

Dr. Collins, of Philadelphia, recommends in the *Med. & Surg. Reporter*, the use of kusso in tenia. He first prescribes at night, on retiring, a full dose of ol. ricini. The following morning he gives an ounce of kusso, in the form of an infusion of a half pint, to be taken at one dose. The teina is expelled before night.

---

**Chloroform and Glycerine.**

Dr. W. Murdock, of New York, recommends the following formula as a convenient mode of administering chloroform: Glycerine, six ounces; chloroform, two ounces. This solution is clear, and not unpleasant in taste or odor. One drachm contains seventeen minims of chloroform.—*Atlanta Medical and Surgical Journal.*

---

**Impotency.**

Prof. D. Hayes Agnew, of Philadelphia, (*Med. & Surg. Reporter*), in a clinical lecture on impotency, after stating that the treatment must be largely moral, and urging the abandonment of onanism or excessive venery, says that there is no better internal treatment than phosphorous and strychnia—1-50 grain of the former, and 1-30 of the latter, made into a pill, and administered three times daily.

---

**Influenza.**

The Philadelphia *Med. & Surg. Reporter*, says that Col. Coolidge, late Medical Inspector U. S. A., who had had experience in several violent epidemics of influenza, used the following formula, and deemed it a *specific* if taken early:

℞

Quiniæ sulphat., gr. vj;
Pulv. ipecac, et opii, comp. gr. x. M.

Ft. cht. j.

**Gleet.**

Dr. R. S. Johnson extols, for cure of gleet, an astringent injection composed of:

℞

Tr. opii. ʒ j;

Tr. catechu, ʒ ss;

Mist. acaciæ, ℥ ij. M.

To be used thrice daily.—*Med. Press & Circular.*

---

**Compound Cathartic Pill.**

The following formula will be found to be an excellent one:

℞

Pulv. jalap, ʒviij;

" aloes soc., ʒvj;

" virg. scammon, ʒiv;

" clocynth,

" gamboge, āā ʒij.

Add syrup q. s., and make 330 pills.—*Druggists Circular.*

---

**Treatment of Dissecting Wounds.**

Dr. John H. Beach, Coldwater, Mich., (*Detroit Review of Medicine*,) having been poisoned by a dissecting wound, was cured through the influence of about fifteen grains of concentrated carbolic acid internally, in about eleven hours medication. Diluting a solution of carbolic acid so that he could swallow it without much irritation of the fauces, he began taking doses equivalent to about twenty drops of the saturated solution every half hour.

---

**Clay Dressings for Small-Pox.**

Dr. E. S. Bunker, of Brooklyn, writes to the *Medical Record*, that during the recent epidemic he used clay dressings for two pretty decided cases of confluent small-pox. Both patients were young women. One, a married lady, æt. 23, (delivered on the second day of a six months' fœtus), made a fair recovery, took cold after getting up, and in a few days died suddenly of empyema and pericarditis; diagnosis confirmed by autopsy. The other, single, æt. 21, had the disease with great violence, recovered rapidly, and is now well. In each case he dusted finely-sifted pipe clay over the face as soon as the pustules became fairly developed. This formed immediately a clean, dry, wholesome scab, abolished the intolerable itching and burning, served apparently as a good absorbent of infectious material, and scaled off during convalescence, leaving underneath a soft, natural integument.

There was no disfigurement in either case.

**Abortive Treatment of Felons.**

Dr. J. H. S., in the *Boston Journal of Chemistry*, has for ten years adopted the plan of applying several coatings of collodion over the finger, or place where the pain is felt, on its first appearance. On drying, the collodion contracts with an even pressure, and if kept on for twenty-four hours the symptoms will usually disappear.—Of late, he has been in the habit of soaking the finger in quite strong solution of carbolic acid, for a few minutes before applying the collodion.—*Oregon Med. & Surg. Reporter.*

**Croup.**

Dr. N. M. Meak, of Callensburg, Pa., writes to the Philadelphia *Med. & Surg. Reporter*, that he has had good success in the treatment of croup, by using the following preparation with the steam atomizer:

℞

Glycerine,

Tinct. myrrh, āā ℨ ijss;

Carbolic acid, grs. ij. M.

To be applied every hour for five or ten mintes at a time.

**On the Use of Lacto-Phosphate of Lime.**

Dr. R. Blacke calls attention, in *The Practitioner* for February, 1872, to the use of this salt in adynamic fevers and in convalescence. The want of success which has generally attended the employment of the phospates, he attributes to the fact that they are given in such quantities that there is not enough lactic acid in the gastric juice to dissolve them. He therefore recommends the administration of lacto-phosphate of lime, which he says is at once an aliment and an article of food, and a medicament of the highest value. It is, moreover, soluble in the secretions of the stomach, and is readily absorbable.

**Quinia and Digitalis in Hemicrania.**

M. Debout, (*The Practitioner*, Feb. 1872, from the *Journal de Medecine*,) has obtained favorable results from the combination of quinia with digitalis in the treatment of migraine. The formula he employs is, sulphate of quinia, forty-six grains; powdered digitalis, twenty-two grains; of syrup, q. s., to be made into thirty pills, of which one is to be taken every evening. M. Gauchet states that he also has had frequent opportunities for treating hemicrania in this manner. In old standing cases it is occasionally ineffectual. He obtained the best results in those cases where the attacks occurred at the menstrual periods.

**New Treatment for Wens.**

Dr. A. J. Perkins says, in the *Georgia Medical Companion*, that the hypodermic syringe is an effectual instrument in the cure of those encysted tumors of the scalp usually called wens, where the patient objects to the knife. It is to be charged with pure tincture of iodine, and the point inserted well into the body of the tumor, and so much of the tincture forced in as the tumor will contain. In a few days the sac shrinks away from the surrounding parts, and may be easily removed.

**Biliary Calculi.**

Dr. John Barclay, in a communication to the *British Medical Journal*, mentions the case of a man who had suffered for twenty-three years from gall stones, and knowing that ethers are solvents of cholesterine, he prescribed chloroform in doses of two or three drops three or four times a day, on the chance of reaching the calculi through the blood. The pain, tenderness, distention and jaundice all disappeared together, and in the eight years which have since elapsed, the patient has never had another attack.

**Chloride of Potassium.**

Dr. Lander has substituted the chloride for the bromide of potassium in the treatment of epileptics with a success which he declares to be identical. He begins with smaller doses; but doses of 75 to 105 grains daily have been borne without inconvenience for months in succession. He states that it is more active, one-sixth of the price, and without the inconvenient secondary effects of bromide of potassium. He believes that, in the stomach, bromide is converted into chloride of potassium; and that, for many reasons, it is desirable to administer it at once in that form.—*Philadelphia Med. & Surg. Reporter.*

**Treatment of Pruritus Vulvæ.**

Those who have had any experience in the treatment of this troublesome affection will learn with interest that Mr. McGrath states, (*Canada Lancet*, Nov. 1871,) that he has found the following, applied by means of a soft sponge after ablution, morning and evening, attended with the most satisfactory and speedy results:

℞
Biborate of soda, ℨ ij;
Hydrochlorate of morphia, gr. xx;
Hydrocyanic acid, ℨ j;
Glycerine, f. ℥ j;
Distilled rose-water, f. ℥ viij.

**Psoriasis.**

R. Lowther, M. D., of Carmel, writes to the *Lancet* that in the treatment of psoriasis he has used for some time a solution of pure carbolic acid, two to four grains to the ounce of water, locally, with the best results. He considers this solution to be more reliable, and much less objectionable to the patient, than the application of pitch ointment. It is often difficult to get patients of the upper classes persuaded to make proper use of the ointment, and on this account much of the failure depends. The patches of psoriasis must be kept constantly moist with the solution of carbolic acid, while the constitutional treatment is not neglected.

---

**New Treatment of Hydrocele.**

The *Gazette Hebdomandairo* contains a paper prepared by M. Monode, describing his method of treating hydrocele and other cerous accumulations, by injecting alcohol either pure or diluted. He first makes a puncture and draws off a drachm or more of the fluid, and then injects the alcohol. The operation is repeated, if necessary. By this method he succeeded in curing three cases of hydrocele, and a cerous cyst of the neck resembling goitere, the injection of a small quantity of alcohol causing a rapid absorption of the entire fluid without inflammation, and without requiring the patients to remain in bed. He suggests the same method for other serous accumulations.—*Pacific Med. & Surg. Jour.*

---

**Lostorfer's Corpuscles.**

Much is being said and written about Syphilitic Corpuscles. Every medical journal we pick up is having its say upon the subject. Dr. Jas. R. Chadwick, in a letter to the Boston *Med. & Surg. Journal*, for May 25th, says that Prof. Stricker has modified his opinion in reference to these bodies, in consequence of finding them in great quantity in a series of preparations from tuberculous and carcinomatious patients. They were also detected in the blood of persons with the following diseases: heart disease, Bright's disease, anæmia and lupus. The development of the corpuscle does not depend, therefore, upon the presence of syphilitic virus, but only upon a condition of the blood resulting from serious constitutional affections. Although they can no longer be offered as pathognomonic indications of syphilis, their presence in the blood may have some weight in guiding us to a diagnosis in questionable cases of exanthemata, periostitis, and the like, *i. e.*, between purely local lesions and similar manifestations of a constitutional malady, since they have been always found in secondary syphilis, even in cases where the patients have been apparently in good health.

---

**Jackson's Cough Syrup.**

According to the *Cincinnati Lancet & Observer*, the formula for this preparation has not been uniform, and therefore the Cincinnati College of Pharmacy has recently presented the subjoined one as a uniform standard:

℞

Fld. ext. ipecac., ʒ ss;

Fld. ext. senegæ, ʒ iij;

Fld. ext. rhei, ʒ iv;

Syr. simplex, ℥ xxxj;

Morphia murias, viij;

Ol. sassafras, gtt. xxxij.

M. fiat mistura.

---

**Chloral in Delirium Tremens.**

Chas. W. Earle, M. D., physician to Washingtonian Home, Chicago, (*Med. Examiner*,) having used chloral in more than fifty cases of delirium tremens, can recommend it for producing sleep. Fifteen grain doses repeated often, until the required result is obtained, seem to give better effect than to give larger doses. Morphine combined with chloral, in some cases, give a better result than either drug given singly. The following formula, of which a teaspoonful may be given every two hours, has given decided benefit:

℞

Chloral hydrat., ʒ ij;

Morphæ acet., gr. ij;

Syrupi zinziberis, ℥ ij. M.

---

**Bisulphite of Soda in Throat Diseases.**

Dr. Tyrrell, in the *Pacific Medical Journal*, commends, as a new remedy in this class of affections, bisulphite of soda, given in large and continuous doses. Diptheria, inflammation of the tonsils and quinsy, though local exhibitions, have their source in poisonous fermentations of the blood, the same as scarlet fever and other zymotic diseases. It is held that the salt prescribed enters into the circulation and retards putrefactive fermentation. Dr. T. failed when he administered it in small doses and in three hour intervals; but when he gave thirty grain doses every hour, day and night, so as to saturate the system with salt, he was almost invariably successful in removing all the severe symptoms in twenty-four hours. He asks physicians to give this medicine a trial, that the curative effects may have more extended proofs.

---

**Cure for the Opium Habit.**

In a recent report on the condition of the English Hospital at Pekin, China, the attending physician gives a formula for "anti-opium pills." This remedy is composed of extract of hyoscyamus or henbane, extract of gentian, camphor, quinine, cayenne peper, ginger and cinnamon, with castile soap and syrup to form the mass, and liquorice powder to form the coating. The efficacy of these pills in overcoming the opium habit, and in preventing the suffering on giving up the use of that poison, is stated to have been proved in numerous cases. The native remedies it is said contain opium in some form, and most frequently the ashes of opium already smoked, and consequently are inefficacious, it being as difficult to discontinue the use of the medicine as of the drug itself.—*Medical and Surgical Reporter.*

---

**Chloral in Venerial Ulcers.**

An Italian physician, Dr. Mancesco Accettilla, uses chloral hydrate as a local application in the cure of venerial ulceration. The solution he employs is as follows:

℞

Chloral hydrate, gram. v;

Aqua distil., gram. xx. M.

This applied with a pencil to the ulcerated surface, slightly cauterizes, without producing discomfort to the patient. After two or three applications, all unhealthy appearance at the bottom of the ulcer disappears, and the sores present a bright redness which soon produces the most healthy granulations.—*Gazette Med. Ital. Lomb.* From the *Boston Med. & Surg. Reporter.*

---

**Xylol.**

This hydrocarbon is likely to become of great importance if its application in cases of small-pox is really followed by such good results as have hitherto been obtained at Berlin.

The Berlin *Klinische Wochenschrift* states that Dr. Zuelzer, Senior Physician at the Charité Hospital, had there administered xylol in cases of small-pox with the most complete success. It is given in doses of from 3 to 5 drops for children, 10 to 15 drops for adults, every hour to every three hours. It is harmless, because as much as a teaspoonful at a time has been taken. The most convenient form of taking it is in capsules, as already supplied by a Berlin firm, and containing 3, 5, 8 and 12 drops each.

The specific action is not yet clearly defined, but early information on this point is promised. The theory at present is, that xylol is taken up by the blood, and acts as a disinfectant.

The absolute purity of the xylol is important, as toluol and other analagous compounds do not possess this peculiar action, and it seems there are some practical difficulties in obtaining xylol absolutely pure.

Pure xylol is colorless, it has a faint odor, somewhat like benzol, but diffierent; boiling-point 139° C., specific gravity 866.

---

**Carbolic Acid in Rubeola.**

Dr. T. J. Williamson has found much advantage from the use of Carbolic Acid in malignant form of measles. In one case his prescription was as follows:

℞

Syrp. Scillæ, c. f. ℥i.

Syrp. Ipecac.,

Tinct. Lobelia Inf. āā. f. ʒj.

Carbolic Acid, gtt. xvj. M.

S. Teaspoonful every hour.

And as a wash for mouth—

℞

Carbolic Acid, ℈ii.

Chlo. Potass. ℈i.

Mellis desp. ℥ss.

Aq. Camph. f. ℥ii. M.

S. Gargle the mouth every three or four hours.

---

**Retention of the Urine.**

Dr. Cazenave, of Bordeaux, (*Jour. de Med. et de Chir.*, May, 1871, quoted in *Med. & Surg. Reporter*,) says that during twenty years, the following simple expedient has never failed in giving relief in retention of urine.

He introduces into the rectum a piece of ice of the form of an elongated oval about the size of a chestnut, which he pushes up beyond the sphincters, and renews every two hours.

Almost always, in an hour and a half or two hours, at longest, urethral spasm ceases, a certain quantity of urine is passed, and the bladder is emptied without effort by the patient. If in rare and exceptional cases this does not take place, he again introduces pieces of ice into the rectum, and also places broken ice from the anus up to end of the urethra, until the urine flows, which it infallibly does. When there is difficulty in making water, occasioned by prostatic hypertrophy, the good effects of the ice are rather longer in coming on, but almost always are produced. In short, in these circumstances (stricture and prostatic hypertrophy), the sedative effects are so well marked, thanks to the effects of the ice, that the introduction of bougies and sounds into the bladder and urethra is always rendered easy to practiced surgeons, and hardly any pain is felt.

---

**Compound Powder of Liquorice.**

The want of a mild but effective aperient, of convenient form and without any of the disagreeable concomitants, induces Dr. David Page to call attention in *The Practitioner*, for May, to the compound liquorice powder of the Prussian Pharmacopœia first introduced into practice in Scotland, by Dr. J. Warburton Begbie. It is composed of the following constituents, so prepared as to formw hen incorported, an almost impalpable powder.

℞
Senna leaves,
Liquorice root, āā, ℥vj;
Fennel seed,
Sulphur, āā, ℥iij;
Refined sugar, ℥xviij. M.

The usual dose is a small teaspoonful at bed time, in water, with which it is easily mixable, forming an agreeable draught. Children readily take it, with the belief that it is sweetmeat. It will be found especially useful in the treatment of constipation resulting from atony of the bowels.

---

**Gastric Juice in Cancer.**

In the *Clinic* for Feb. 10th, may be found a report of a series of experiments conducted by Dr. Stohr of Wurzburg. The investigations were made with gastric juice obtained from the stomachs of dogs, either by the establishment of fistulæ, or by first killing the animal and opening the stomach without delay; the juice was also secured from owls and crows, by introduction of sponges into the œsophagus.

The application of this juice to the ulceration was made either by the use of a brush or by pledgets of cotton kept saturated. Experiments were performed upon primary syphilitic ulcers and upon cancer. Very little pain attended the application. Examination of the sore three or four hours after the treatment showed upon the surface a layer of grayish-white, semi-transparent tissue of gelatinous aspect, moderately adherent; this layer thickened after the repetition of the application, and there was developed, after a few days, a decided tendency to healthy cicatrization.

The author thinks it is, in a certain sense, a caustic; it digests the tissues to which it is applied, transforming them into pepsine. It quickly supplants a phagadenic sore by luxurient granulations.

---

**Cases Treated with Chloral.**

Dr. M. More Madden, of Dublin, in an article to the *Journal of Materia Medica*, recommends the use of chloral hydrate in a number of diseases. We make a few extracts from the article as follows—

ODONTALGIA.—A woman æt. 30, confined two days previously, was in great pain from violent toothache, which has prevented sleep since her confinement. Gave her 15 grs. of chloral in draught, giving her immediate ease, and within a few minutes produced sleep which lasted 3 hours.

INSOMNIA WITH THREATENED PUERPERAL MANIA.—Woman, æt. 40, unmarried, delivered of second child, after natural labor. Was in great distress of mind; did not sleep for three nights; talked wildly; got out of bed and insisted on going home the day after her confinement; manifested an aversion to the child. Gave her 50 grs. of chloral, and a half hour after she fell asleep, and slept for the first night since her admission into the hospital. Next morning she was much better.

INSOMNIA.—A woman æt. 26, suffering from debility after confinement and hemorroids. A very nervous, excitable woman, unmarried, and in despondent frame of mind. Has not slept for three nights; when she drowses is awakened by fearful dreams; manner abrupt and peculiar; appears not far from being insane; tongue dry and covered with white fur; pulse 104; countenance pale and anxious; complains of great headache and palpitation.

℞

Chloral hydratis, ʒj.;

Tinct. valerianæ ammoniat, ℥iij;

Infusi valerianæ, ℥vj. M.

Sig: ℥j. every two hours.

After taking the second dose of the mixture she went to sleep; passed a good night, and awoke decidedly better.

DELERIUM TREMENS.—Was sent for to see a young man who had been drinking hard for several days, but had stopped drinking three days ago, and had not slept since. Was in a state of great nervous excitement; his face pale and anxious; skin cool and moist; pulse quick, small and compressible; tongue furred and tremulous. His wife informed me that she, her servant and two friends, had passed the last two nights keeping him in bed, from which he was endeavoring to escape. He had taken no nourishment whatever for three days; complains of great thirst, which he has been striving to slake by copious draughts of cold water, vomited as soon as swallowed. Ordered milk and soda water in small quantities, and the following draught:

℞
Hydratis chlorali,
Potassi bromidi, āā. grs. xxx;
Aquæ camphoræ, f. ℥ ij. M.

This was given at 11 P. M., when his pulse was 106. In 15 minutes his pulse continued as before; in 30 minutes pulse 102—fell asleep. He slept all night; awoke more tranquil and took some soup. Next night he had 20 grs. of chloral; slept well and rapidly convalesced.

RIGIDITY OF OS UTERI.—A primipara, æt. 22, admitted in labor, has been in labor since 10 P. M., on 26th. On 27th, at 11 P. M., os size of a florin, thick and rigid. On 28th, at 8 P. M., the os being still in same condition, though, in the meantime, she had been treated in the ordinary manner, by warm baths, opiates, and solution of tartar-emetic, I resolved to try the effects of chloral, and ordered the following draught, which was given at 8:50 P. M., her pulse then being 100:

℞

Hydratis chlorali, xl;

Mel. despumat. ʒ j;

Aquæ cinnamomi, ℥ j. M.

Sig: Fiat haustus, statim sumend.

At 9 P. M., asleep, pulse 90. 9:10 P. M., pulse 88, still asleep. 9:38 P. M., roused for a moment, had a pain, and settled down again. 10 P. M., still asleep. For next quarter of an hour was restless and in pain; was ordered a second draught with 20 grs. of chloral; in all, one drachm in one hour and twenty mintues. After taking draught, she at once fell asleep again. At 3 A. M., she woke up, and at 4:30 A. M., she was delivered.

